# The recent Physics and Chemistry Nobel Prizes, AI, and the convergence of knowledge fields

**DOI:** 10.1016/j.patter.2024.101099

**Published:** 2024-11-25

**Authors:** Charles H. Martin, Ganesh Mani

**Affiliations:** 1 Calculation Consulting, San Francisco, CA, USA; 2 Carnegie Mellon University, Pittsburgh, PA, USA

## Abstract

This article examines the convergence of physics, chemistry, and artificial intelligence (AI), highlighted by recent Nobel Prizes. It traces the historical development of neural networks, emphasizing interdisciplinary research’s role in advancing AI. The authors advocate for nurturing AI-enabled polymaths to bridge the gap between theoretical advancements and practical applications, driving progress toward artificial general intelligence (AGI).

## Main text

### Introduction

Recent developments have marked an exciting period for AI. The 2024 Nobel Prize in Physics was awarded to John Hopfield and Geoffrey Hinton for their foundational work in AI, while the Chemistry Prize went to David Baker, Demis Hassabis, and John Jumper for their use of AI to solve the protein-folding problem, a *50-year grand challenge problem in science*.

With AI recognized in connections to both physics and chemistry, machine learning (ML) practitioners may wonder how these sciences relate to AI and how these awards might influence their work.

### Historical development of neural networks

By examining the history of AI development, we can better understand the interconnections between computer science, theoretical chemistry, theoretical physics, and applied mathematics. This historical perspective illuminates how foundational discoveries and inventions across these disciplines have enabled modern machine learning with artificial neural networks.

The earliest work in the field of neural networks is attributed to McCulloch and Pitts, who, in 1943, developed a model representing neurons using simple binary logic. Their work laid the foundation for modern neural networks by demonstrating that neurons could implement basic logical operations like *AND*, *OR*, and *NOT*. However, they did not address mechanisms for learning or storing long-term memories in networks of biological neurons. Many computer scientists are familiar with these early logical models, which serve as the conceptual basis for today’s complex neural decision-making processes.

Donald Hebb, in 1949, proposed that neurons firing repeatedly together or in close temporal proximity strengthen their connections, a principle now known as “Hebbian learning.” In the late 1950s, Frank Rosenblatt made significant strides in neural network research by coining the term “perceptron” and developing both theoretical models and hardware implementations. Rosenblatt’s perceptrons introduced non-binary inputs and trainable weights between neuronal units, adding flexibility to neural network designs.

However, in 1969, Marvin Minsky and Seymour Papert’s influential book *Perceptrons* highlighted a crucial limitation: networks without trainable hidden units were incapable of solving non-linearly separable problems such as the exclusive *OR* (*XOR*) function or detecting spirals in satellite images. This revelation, while initially discouraging, ultimately spurred the AI community to focus on the challenge of training networks with hidden units, leading to the development of the backpropagation algorithm, with significant contributions from Geoffrey Hinton and others. Concurrently, researchers in theoretical physics, chemistry, and applied mathematics were exploring neural network models of real-world phenomena, including the behavior of spiking biological neurons, further enriching the field’s interdisciplinary nature.

We can trace early efforts to model real-world neurons back to Jack Cowan and Shun’ichi Amari, whose research generalized the Volterra predator-prey equations, originally for animal population dynamics, and applied them to spiking neurons. For decades, Cowan has advanced the statistical mechanics of neurons, while Amari’s pioneering work in neural network dynamics and learning algorithms continues to influence modern ML.

### Key breakthroughs and challenges

A conceptual breakthrough came with John Hopfield’s work, which introduced a simplified model of neurons and connected it back to the Ising model from theoretical physics and chemistry.

Hopfield’s classic paper in 1982^1^ introduced the Hopfield associative memory model, which became foundational in the study of neural networks. The model demonstrated how a network of neurons could store and retrieve patterns through energy minimization, with the system’s dynamics governed by an energy function. This connection to energy allowed the model to settle into stable states that corresponded to stored memories, making it a key link between neural network theory and spin glass theory from theoretical physics.

Theoretical physicists, familiar with spin glass theory, developed new theories of learning that offer a perspective competing with the more familiar statistical learning theory from computer science, providing significant insights into contemporary problems. These insights have broad implications today for understanding large language models (LLMs), diffusion models, and more. For example, the “double descent” phenomenon in deep neural nets (DNNs) was first identified in the theoretical physics literature while studying perceptron learning models.

Hopfield’s associative memory model exhibited a phenomenon similar to what we now call hallucinations in neural networks. In neuroscience, this model is even applied to study visual hallucinations. Hinton and Terrence Sejnowski combined ideas from simulated annealing with Hopfield’s spin-glass model to propose the Boltzmann machine.^2^^,^^3^ This approach allowed for unsupervised learning, creating internal models of regularities in training data without requiring input-output pairs. This work drew more heavily on analogies to physical and thermodynamic systems.

On the supervised learning front, to overcome the Minsky and Papert critique, Hinton and many others were continuing the work on multi-layered neural networks with hidden units. The two-volume *Parallel Distributed Processing* series, edited by David Rumelhart and James McClelland in 1986, became the *sine qua non* for young researchers and students exploring the field of neural networks, establishing itself as the essential reference for connectionist studies. Hinton, along with David Rumelhart and Ronald Williams, popularized the backpropagation approach within the AI community in the 1980s^4^ Hinton’s persistence in this line of research, when many others had abandoned it as suitable only for toy problems, was eventually vindicated as computational power exploded and new algorithmic variants were developed.

Of course, today’s neural networks are much more sophisticated than the original Hopfield associative memory or the popular backpropagation-based models of the 1980s. While deep learning has made tremendous strides in solving many previously insurmountable problems in machine learning and artificial intelligence, we still lack even a basic theory to explain why it works. Practitioners often struggle with the chaotic and unpredictable behavior of LLMs when deployed in production. However, progress has been made by revisiting older models from statistical physics. For example, the heavy-tailed self-regularization (HTSR) theory developed by Martin and Mahoney in 2021^5^ can detect if the individual layers of a neural network have converged without needing to examine the training or test data.

**Figure 1:**
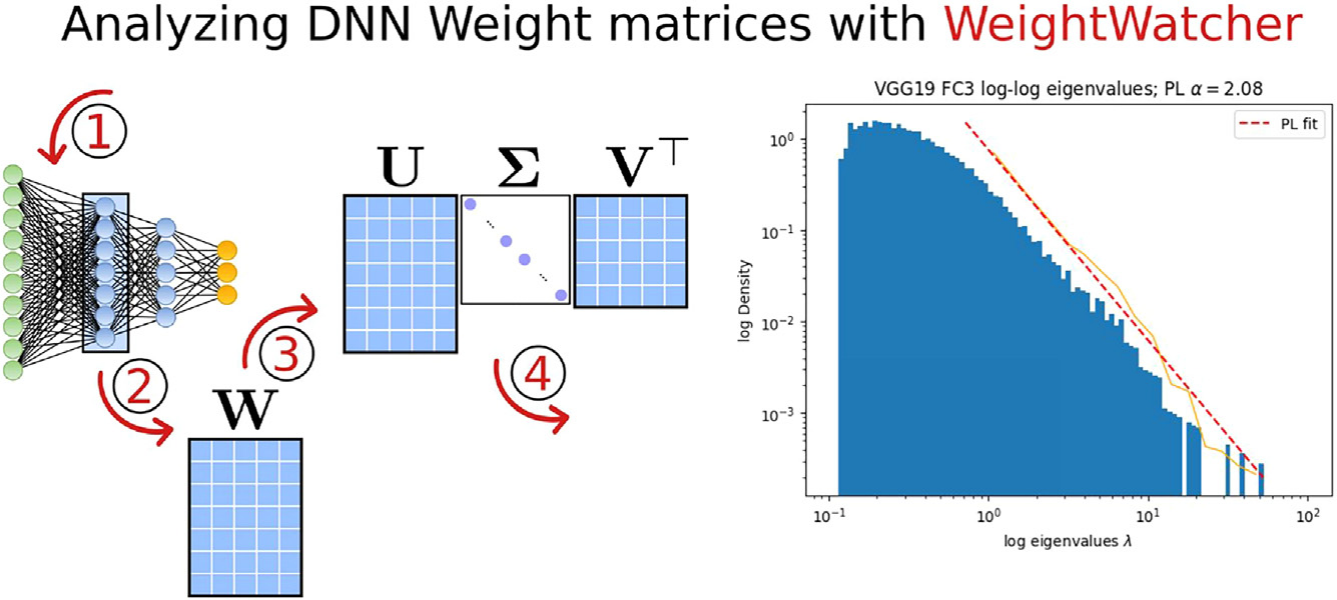
Through the WeightWatcher looking glass

The HTSR theory is implemented in the open-source, data-free diagnostic tool WeightWatcher. Using WeightWatcher, we can look inside modern DNNs and observe that the best-performing models display a remarkable universality, as predicted by the HTSR theory, where the tail of the empirical distribution of the eigenvalues of the layer weight matrices converge to a power law (PL) distribution, with a PL exponent alpha ≈ 2. In Figure 1, we show a histogram of the eigenvalues on a log-log plot for the weight matrix of the last layer of VGG19 and the fit to a PL distribution.

This very common behavior suggests that a more fundamental as yet unknown universal computational process governs the training of DNNs. While we do not understand the origin of this seemingly universal phenomenon, its observation suggests that a new physics is yet to be discovered that may incorporate some elements of statistical mechanics but in a fundamentally new way.

### Engineering preceding science

It is not uncommon for engineering to precede scientific understanding. For example, the development of steam engines, cars, boats, and locomotives advanced well before we had a deep grasp of their underlying dynamics. Early steam engines were prone to explosions, causing fires and even sinking ships. It was only with the advent of thermodynamics that we truly came to understand how to control and optimize these complex machines. This scientific foundation eventually led to the refinement of the combustion engine and paved the way for advanced technologies like today’s *SpaceX* rockets. The rapid development and widespread adoption of cryptocurrencies further exemplify how engineering can outpace scientific understanding. These digital assets have revolutionized financial transactions and sparked a global phenomenon, all before a comprehensive theoretical framework for their behavior and impact could be established.

The other recent Nobel Prize—in Chemistry—illustrates that, even without a complete theoretical understanding, AI can help solve some of the most challenging scientific problems. By cracking the protein-folding problem, John Jumper and Demis Hassabis have paved the way for groundbreaking discoveries in biology and medicine. Their work with AlphaFold^6^ demonstrates the transformative potential of AI, not only in solving complex puzzles but also in accelerating advancements across numerous scientific fields.

At first glance, it may seem AlphaFold appeared out of the ether, but in fact, the protein-folding problem has a long history connected to theoretical physics, chemistry, and AI. Indeed, Jumper’s thesis work was a hybrid physics-AI model (trained with Hinton’s contrastive divergence algorithm for restricted Boltzmann machines [RBMs]) that could address both protein folding and dynamics. The early statistical mechanics approach incorporated empirical data using AI by averaging over fast side-chain motions. This was achieved by fitting parameters to Protein Data Bank (PDB) data using an algorithm similar to an RBM. However, these calculations were enormously computationally intensive, requiring the world’s largest supercomputers. By combining theory, experimental data, and AI, Jumper’s early work laid crucial groundwork that eventually contributed to the spectacular breakthroughs seen in AlphaFold.

Many of the early pioneers of neural networks recognized the potential of massive parallelism in computational models.^7^^,^^8^ However, the full realization of this concept remained elusive because of the technological constraints of the era. The computing hardware of the time, with its relatively slow CPUs and absence of specialized processors like GPUs, posed significant limitations on implementing truly parallel architectures at scale. Despite these hurdles, the field made crucial strides in theoretical foundations. The introduction of distributed encoding marked a paradigm shift, moving away from localized representations to more robust and flexible distributed patterns of activation across neural networks. This approach allowed for more nuanced and context-sensitive information processing. Equally transformative was the development of learning through backpropagation of errors, a method that enabled networks to adjust their internal parameters based on the discrepancy between predicted and actual outputs. This algorithm, though computationally intensive, provided a powerful mechanism for training multi-layer networks, laying the groundwork for the deep learning revolution that would unfold decades later when hardware capabilities finally caught up with the theoretical vision of these early researchers.^9^

As we reflect on the historical challenges and breakthroughs in neural networks, it is crucial to consider how these advancements have shaped our understanding of the world around us. This evolution in computational thinking has led us to reconsider the very building blocks of our universe, expanding our perspective beyond traditional scientific boundaries. This expanded perspective invites us to explore a new conceptualization of contemporary building blocks that intersect with our understanding of nature.

### Evolution of nature’s building blocks

The fundamental architecture of our world can be conceptualized as a triumvirate of primary building blocks or foundational “ABCs”: “atoms,” the basic units of matter; “bits,” the smallest indivisible units of information; and “cells,” the foundational elements of life. Our quest to understand nature’s core constituents has undergone significant evolution, transitioning from the tangible realm of atoms and the biological domain of cells to the abstract yet omnipresent realm of bits. This transformation represents a paradigm shift in scientific investigation wherein information has emerged as a central focus, complementing and frequently intermingling with our comprehension of matter and life.

The integration of bits as a fundamental unit of information has catalyzed a new era of interdisciplinary research and explorations, effectively diminishing traditional barriers between scientific disciplines. This digital revolution has facilitated innovative methodologies for addressing long-standing questions across multiple fields. For example, bioinformatics combines computer science and molecular biology to analyze the human genome, while computational linguistics employs statistical models to decipher the intricacies of human language. In the realm of climate science, big data analytics and machine learning algorithms process extensive datasets of environmental information, resulting in enhanced predictive accuracy and deeper insights into global climate phenomena. Many more contemporary examples abound, demonstrating how the concept of bits functions as a unifying principle, interconnecting diverse disciplines and turbocharging innovative solutions to complex, multifaceted challenges.

### Convergence of poets and quants

Humanities, liberal arts, and traditionally quantitative fields like STEM have often been separate domains of education and practice. While some interaction has always existed, we are now witnessing increased blending, thanks in part to technologies like language models and chatbots. These tools facilitate the creation of simpler versions of complex technical texts and allow for the analysis and visualization of qualitative data from the humanities. This convergence encourages diverse global teams to learn through real experiences and problem-solving, such as in many of the project-based courses—with an experiential learning from AI and data motif—taught by one of the authors (G.M.).

Tools such as Google’s *NotebookLM* facilitate easy generation of summaries of complex documents with a single click. This allows people to quickly consume information, even in podcast form, providing them with concise overviews.

### Call to action

As we move forward, it is crucial to recognize the convergence of different approaches, such as backpropagation-based deep learning and the physics-inspired Boltzmann machines, in shaping modern AI systems based on generative AI.

This interdisciplinary approach is not just beneficial but essential for addressing the many complex challenges that lie ahead. We need to harness the momentum of current advancements while remaining grounded in practical realities. The rapid progress of AI across diverse sectors presents both unprecedented opportunities and significant challenges. To bridge the gap between hype and tangible development, we must cultivate a new generation of interdisciplinary thinkers: the AI-enabled polymaths of our time.

These modern-day Leonardo da Vincis will be crucial in developing practical learning theories that can be immediately applied by engineers, propelling us toward the ambitious goal of artificial general intelligence (AGI). This calls for a paradigm shift in our approach to scientific inquiry and problem-solving, one that embraces holistic, cross-disciplinary collaboration and learns from nature to understand nature. By breaking down silos between fields and fostering a culture of intellectual curiosity that spans multiple domains, we can unlock innovative solutions to complex global challenges such as climate change. It is through this synthesis of diverse knowledge and perspectives, catalyzed by AI, that we will forge a path to meaningful progress and realize the full potential of our technological aspirations.

As AI continues to advance, the frontier of consciousness emerges as a tantalizing challenge, raising questions about whether it can be fully explained through the interplay of atoms, bits, and cells or if a new, as-yet-undiscovered element is required. Recent work by Blum et al. proposes a novel “conscious Turing machine” model,^10^ suggesting that consciousness might be understood and potentially replicated through computational processes, thus bridging the gap between traditional AI and the enigma of self-awareness. This frontier exemplifies the need for the interdisciplinary approach we advocate, combining insights from computer science, neuroscience, philosophy, physics, and chemistry to tackle one of the most profound questions in AI and human understanding.

## Acknowledgments

We thank emeritus CMU Professor Scott E. Fahlman, of smiley fame :-) and a doyen of the neural network field, for his helpful feedback. We note that Hinton was part of the CMU faculty in the 1980s, collaborating with Fahlman during that time.

## Declaration of interests

C.H.M. is a principal of Calculation Consulting. The open-source tool WeightWatcher affiliated with his firm is used to illustrate a technical point herein. G.M. is on the advisory board of this journal, *Patterns*. He is also involved in consulting and holds various other advisory board positions; none of these activities create a conflict with this opinion.

## Declaration of generative AI and AI-assisted technologies in the writing process

During the preparation of this work, the author(s) used Perplexity.ai to refine the sentence structure of certain paragraphs. After using this tool, the author(s) reviewed and edited the content as needed and take(s) full responsibility for the content of the publication.
